# Linkage between IL-23 and coronary arterial lesions in pediatric patients with Kawasaki disease

**DOI:** 10.3389/fped.2026.1769445

**Published:** 2026-02-23

**Authors:** Yi Wei, Siqi Feng, Jinhui Wu, Penghui Yang, Ya Su, Qijian Yi

**Affiliations:** 1 Department of Cardiology, National Clinical Research Center for Children and Adolescents’ Health and Diseases, Ministry of Education Key Laboratory of Child Development and Disorders, Children’s Hospital of Chongqing Medical University, Chongqing, China; 2 National Clinical Key Cardiovascular Specialty, Chongqing, China; 3 Key Laboratory of Children’s Vital Organ Development and Diseases of Chongqing Municipal Health Commission, Chongqing, China

**Keywords:** coronary arterial lesions, IL-23, intravenous immunoglobulin, Kawasaki disease, pro-inflammatory

## Abstract

**Background:**

Coronary artery lesions (CALs) in Kawasaki disease (KD) are thought to arise from aberrant immune activation and an amplified inflammatory cascade triggered by an unidentified etiologic factor. Interleukin-23 (IL-23)—a pivotal modulator of chronic inflammatory responses and immune-mediated vascular damage—has lately garnered interest regarding its putative role in cardiovascular pathological processes

**Aim:**

To explore the correlation between circulating IL-23 concentrations and the occurrence of CALs in pediatric patients with KD.

**Methods:**

Peripheral blood samples were obtained from 103 pediatric patients with KD prior to administration of intravenous immunoglobulin. Using Enzyme-Linked Immunosorbent Assay (ELISA), we quantified circulating cytokine levels in a total of 211 study participants, who were stratified into four distinct cohorts: 47 KD cases with coronary artery lesions, 56 cases without vascular involvement, 58 febrile controls, and 50 healthy controls.

**Results:**

Serum IL-23 concentrations were markedly elevated in children with KD [279.69 pg/mL (132.67–693.32)] compared with both febrile controls [161.02 pg/mL (81.50–338.60)] and healthy controls [132.41 pg/mL (61.74–274.28)] (*P* < 0.001), indicating a disease-specific elevation. Among KD patients, 47 (45.63%) developed CALs. The KD individuals presenting with CAL (KD-CALs) group exhibited markedly higher IL-23 levels [395.76 pg/mL (221.62–1,217.19)] compared with KD individuals without CAL (KD-NCALs) [222.81 pg/mL (100.18–388.58), *P* < 0.001], accompanied by higher Erythrocyte Sedimentation Rate (ESR) and increased Interleukin-6 (IL-6), matrix metalloproteinase-1 (MMP-1), vascular endothelial growth factor (VEGF) levels. IL-23 displayed significant positive associations with multiple inflammatory indices, including white blood cell count (WBC), C-reactive protein (CRP), IL-6, Interleukin-10 (IL-10), Interleukin-17A (IL-17A), MMP-1, and VEGF. Receiver operating characteristic (ROC) analysis showed that IL-23 effectively discriminated KD from controls [area under the curve (AUC) = 0.71, cutoff = 202.3 pg/mL, sensitivity = 66.0%, specificity = 68.0%] and KD-CAL from KD-nCAL (AUC = 0.69, cutoff = 661.2 pg/mL, sensitivity = 42.6%, specificity = 87.5%).

**Conclusion:**

Elevated serum IL-23 is associated with heightened inflammatory activity and the presence of coronary artery lesions in KD, suggesting that IL-23 may contribute to CAL pathogenesis and represent a potential biomarker of vascular involvement.

## Introduction

Kawasaki disease (KD) denotes an acute-onset, self-resolving systemic vasculitic disorder that primarily impacts infants and pediatric patients aged below five years (1). Formation of coronary artery lesions (CALs) alongside coronary artery aneurysms is the most clinically consequential complication of this condition (2). Although the precise mechanisms underlying CAL formation remain incompletely elucidated, current evidence suggests that an unidentified trigger induces immune dysregulation, leading to an exaggerated inflammatory cascade (3). Notably, KD is characterized by perturbations in T-cell subsets and pronounced upregulation of pro-inflammatory mediator—encompassing Interleukin (IL)-6, Interleukin-17A (IL-17A), and Interleukin-10 (IL-10)—over the early and intermediate stages of the disease (4–6).

Interleukin-23 (IL-23) acts as heterodimeric cytokine molecule within the Interleukin-12 (IL-12) family of cytokines, a family that contains one p40 subunit component shared with IL-12 and one distinct p19 subunit moiety exclusive to IL-23 among all family members (7). Dendritic cells and macrophages serve as the primary cellular sources of this particular cytokine molecule, secreting it predominantly following their activation (8, 9). Extensive evidence identifies IL-23 as a central mediator in autoimmune and inflammatory disorders, including rheumatoid arthritis, asthma, and atherosclerosis. Via the induction of T helper 17 cell differentiation alongside functional activation, IL-23 triggers the subsequent synthesis of downstream inflammatory mediators, encompassing IL-17, IL-6, Interleukin-22 (IL-22), and tumor necrosis factor-α (TNF-α) (10, 11). Despite its recognized role in vascular and immune-mediated inflammation, the contribution of IL-23 to KD pathogenesis has not been fully established (12, 13). Given these observations, we measured circulating IL-23 levels in individuals with KD and explored linkages among this cytokine as well as pro-inflammatory mediators—including IL-17A, IL-10, IL-6, matrix metalloproteinase-1 (MMP-1), and vascular endothelial growth factor (VEGF)—with the aim to elucidate whether IL-23 contributes to the inflammatory cascades underlying KD pathogenesis and the formation of CALs.

## Materials and method

### Subject population

Patients diagnosed with KD were recruited from the Children's Hospital of Chongqing Medical University, Chongqing, China. A total of 103 children with KD (63 males, 40 females; Age, M (Q₁, Q₃): 1.90 (1.38, 2.75) who fulfilled the diagnostic benchmarks formulated by Japan's Kawasaki Disease Research Committee (14) were enrolled. Additionally, 50 healthy children (39 males, 11 females; Age, M (Q₁, Q₃): 2.47 (1.56, 3.36) served as normal controls (NC group), and 58 febrile children with acute infectious illness (39 males, 19 females; Age, M (Q₁, Q₃): 2.38 (1.20, 4.49) served as febrile controls (FC group). The infectious etiologies in the febrile control children included acute upper respiratory tract infections, community-acquired pneumonia, urinary tract infections, acute gastroenteritis, among others. Their body temperature range was comparable to that of the KD children, with all presenting acute fever (body temperature ≥38.5 °C). However, Kawasaki disease and other autoimmune disorders were ruled out by clinical and laboratory examinations.

Individuals with KD were further subclassified according to coronary artery involvement status into two cohorts: individuals presenting with CAL (KD-CALs, *n* = 47) and individuals without CAL (KD-NCALs, *n* = 56). CALs were defined as a luminal internal diameter of over 3.0 mm in pediatric patients younger than 5 years old, in excess of 4.0 mm in children aged 5 years and older, or alternatively a vascular dilation of no less than 1.5 times the diameter of a neighboring vascular section—criteria established via echocardiographic assessment (15). Echocardiographic examinations were conducted either prior to intravenous immunoglobulin (IVIG) administration or within the first two weeks following disease onset. Blood samples from all pediatric subjects were collected during the acute febrile phase. Specifically, samples from the KD group were obtained on days 3–5 (median day 4) after disease onset and prior to IVIG infusion. Samples from the FC group were collected on days 3–5 (median day 4) of the febrile illness, while samples from the NC group were obtained under healthy conditions. Serum specimens were preserved at −80 °C until laboratory analysis was performed.

### Quantification of cytokine concentrations and clinical laboratory indices

Circulating concentrations of IL-23, IL-6, IL-17A, IL-10, MMP-1, and VEGF were determined via commercially sourced Enzyme-Linked Immunosorbent Assay (ELISA) kits (RayBiotech, USA), in strict adherence to the standardized experimental protocols. Routine clinical laboratory indices—encompassing white blood cell count (WBC), red blood cell count (RBC), platelet count (PLT), C-reactive protein (CRP) levels, Erythrocyte Sedimentation Rate (ESR), and procalcitonin (PCT) concentrations—were extracted from the electronic medical records of the participating hospital. This research protocol underwent review and approval by the Ethics Review Board of Chongqing Medical University's Children's Hospital. Written informed consent was secured from each of the legal custodians acting on behalf of all study participants prior to their enrollment in the research.

### Statistical analysis

Continuous variables were expressed as the mean ± standard deviation (SD), whereas categorical variables were reported as frequencies and percentages (*n*, %). Intergroup comparisons were conducted via one-way analysis of variance (ANOVA), with subsequent unpaired two-tailed t-tests applied for *post-hoc* assessments when deemed appropriate. We assessed the correlations between cytokine concentrations and clinical laboratory parameters via Pearson's product-moment correlation coefficient analysis. Statistical significance was defined as a two-tailed *P*-value below 0.05.

## Results

### Patient demographics

We recruited 103 pediatric patients with KD, 50 normal control subjects (NC), and 58 febrile control individuals (FC) for the present study. Among the KD cohort, 63 (61.17%) were male and 40 (38.83%) were female, while in the NC and FC groups, males accounted for 78.00% (*n* = 39) and 67.24% (*n* = 39), respectively. Statistical analysis of sex distribution across the three study groups demonstrated no statistically significant disparities (*χ*²=4.31, *P* = 0.116). Participants within the KD cohort exhibited a median chronological age of 1.90 (1.38–2.75), while the NC group presented with a median age at enrollment of 2.47 (1.56–3.36) and the FC group had a median age at study entry of 2.38 (1.20–4.49). Likewise, no marked variation in age was detected across the three study cohorts (*χ*² = 4.09, *P* = 0.13) (Table 1). Such observations indicate that three cohorts were demographically comparable at baseline, thereby validating the robustness of subsequent analytical approaches. Among individuals in the KD cohort, 47 patients (45.63%) presented with CAL, while 56 cases (54.37%) presented without CAL (KD-NCALs).

**Table 1:** Baseline demographic and clinical traits in Kawasaki disease (KD), normal control (NC), febrile control (FC) groups.

Variables	KD (*n* = 103)	NC (*n* = 50)	FC (*n* = 58)	Statistic	*P*
Age, M (Q₁, Q₃)	1.90 (1.38,2.75)	2.47 (1.56,3.36)	2.38 (1.20,4.49)	*χ*² = 4.09^a^	0.13
Gender, *n* (%)				χ² = 4.31	0.116
Male	63 (61.17)	39 (78.00)	39 (67.24)		
Female	40 (38.83)	11 (22.00)	19 (32.76)		
Coronary artery lesions (CAL)	47 (45.63%)				
Incomplete Kawasaki disease(iKD)	56 (54.37%)				

^a^ Kruskal-Waills test, χ², Chi-square test; M, median; Q₁, 1st quartile; Q₃, 3st quartile.

### Elevated circulating IL-23 concentrations in pediatric KD cohort

**Figure 1:**
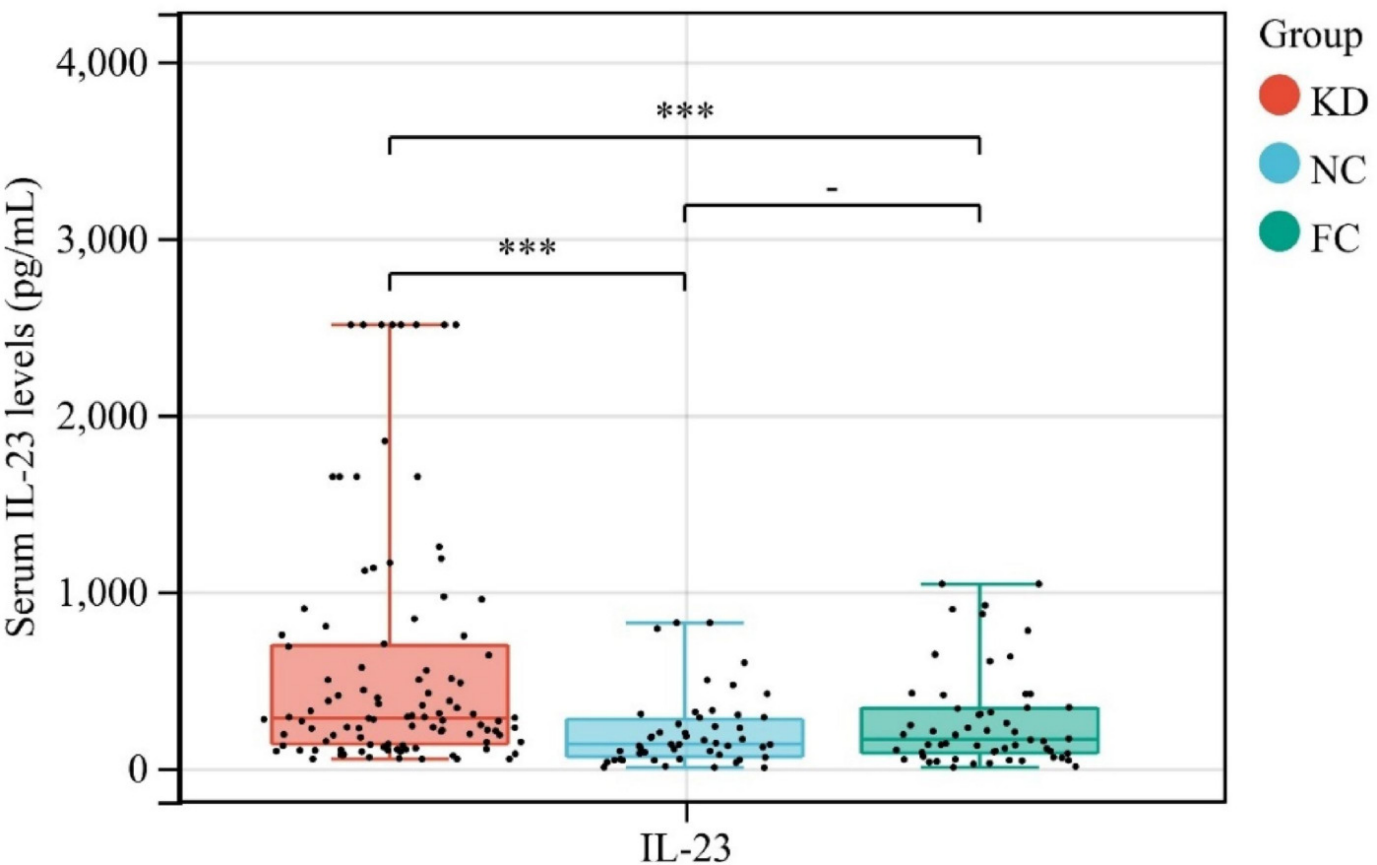
Analysis of circulating IL-23 concentrations across Kawasaki disease, NC and FC groups. ***P < 0.001.

Serum IL-23 levels differed significantly among the three groups (*χ*² = 21.79, *P* < 0.001). Marked variability in circulating IL-23 was observed across the three cohorts (*χ*² = 21.79, *P* < 0.001). As illustrated in Figure 1, children with KD exhibited substantially elevated serum IL-23 concentrations [median 279.69 pg/mL [interquartile range (IQR) 132.67–693.32] compared with both febrile controls [161.02 pg/mL (81.50–338.60)] and healthy subjects [132.41 pg/mL (61.74–274.28)]. These findings indicate that the elevated IL-23 level in KD patients reflects a disease-specific immune response rather than a nonspecific inflammatory reaction.

### Higher IL-23 concentrations in KD children with CALs

**Figure 2:**
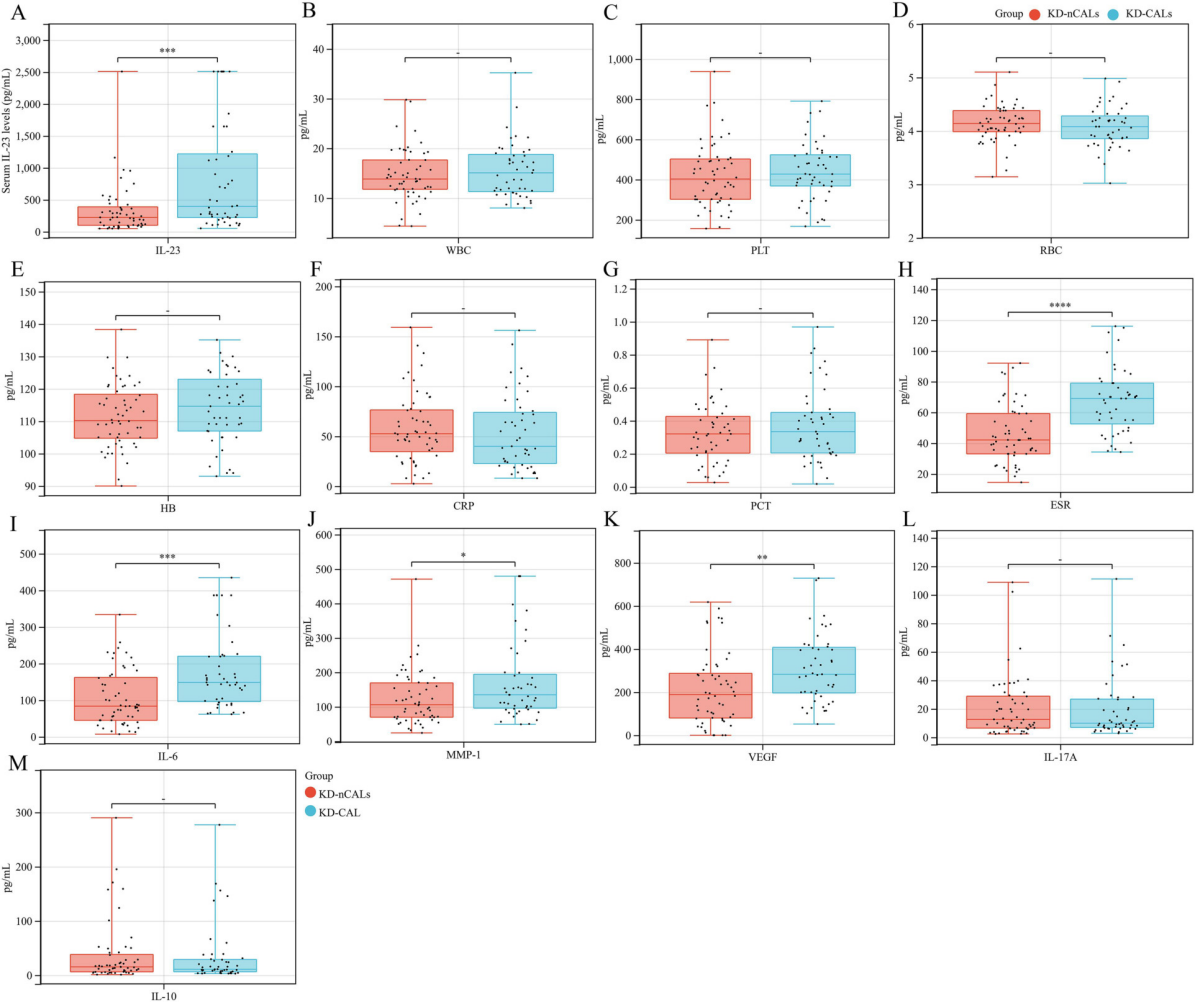
Comparative analysis of Pro-inflammatory mediators and cytokine profiles Among pediatric KD patients who present With versus those without CALs. **(A)** IL-23, **(B)** WBC, **(C)** PLT, **(D)** RBC, **(E)** HB, **(F)** CRP, **(G)** PCT, **(H)** ESR, **(I)** IL-6, **(J)** MMP-1, **(K)** VEGF, **(L)** IL-17A, and **(M)** IL-10. *P < 0.05, **P < 0.01, ***P < 0.001.

Baseline traits, encompassing age and gender distribution, were comparable across the KD-CAL and KD-nCALs cohorts (Table 2). Circulating IL-23 concentrations exhibited a marked elevation among the KD-CAL cohort compared with the KD-nCALs cohort [395.76 picograms per milliliter (221.62–1,217.19) vs. 222.81 picograms per milliliter (100.18–388.58), *P* < 0.001]. Similarly, the KD-CAL group exhibited markedly elevated ESR [69.00 (52.50–79.00) vs. 42.05 (33.00–59.23), *P* < 0.001], IL-6 [148.57 (96.04–219.94) vs. 83.53 (45.00–161.99), *P* < 0.001], MMP-1 [134.45 (95.67–194.22) vs. 105.60 (69.61–169.13), *P* = 0.022], and VEGF [282.83 (196.04–407.64) vs. 188.53 (79.89–286.93), *P* = 0.002]. No statistically significant disparities were detected across the two cohorts in terms of WBC, PLT, RBC, hemoglobin (HB), CRP, PCT, IL-17A, or IL-10 levels (*all P* > 0.05) (Figure 2).

**Table 2:** Baseline demographic characteristics of KD children with and without CALs.

Variables	KD-CAL (*n* = 47)	KD-nCALs (*n* = 56)	Statistic	*P*
Age, M (Q₁, Q₃)	1.75 (1.13, 2.80)	1.90 (1.42, 2.75)	Z = –0.65	0.516
Sex, *n* (%)			χ² = 0.50	0.478
Male	27 (57.45)	36 (64.29)		
Female	20 (42.55)	20 (35.71)		

Z, Mann–Whitney test,; χ², Chi-square test; M, median; Q₁, 1st quartile; Q₃, 3st quartile.

### Associations between circulating IL-23 concentrations with cytokine expression patterns in KD patients

**Figure 3:**
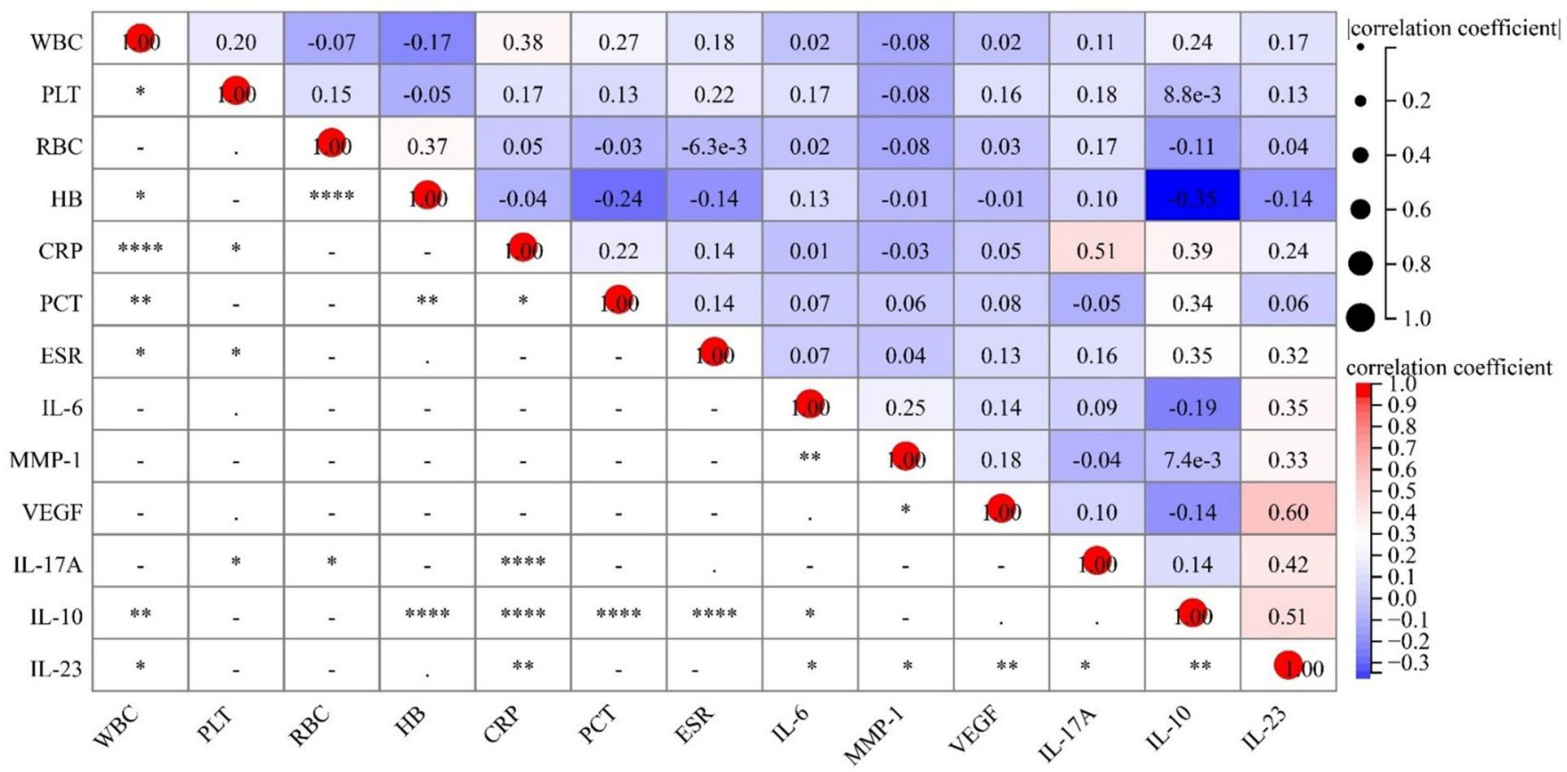
Heatmap of IL-23 correlations with clinical characteristics in KD patients.

To additionally elucidate the association between IL-23 and systemic inflammation in KD, correlation analyses were performed between IL-23 levels and hematological as well as inflammatory biomarkers (Table 3 and Figure 3). Circulating IL-23 exhibited a positive correlation with WBC counts (r = 0.17, *P* = 0.043) and CRP levels (r = 0.24, *P* = 0.025), a finding that points to its association with systemic inflammatory reactions in KD patients. Moreover, IL-23 levels showed significant positive correlations with multiple cytokines, including IL-6, IL-10, and IL-17A, pointing to a strong linkage of circulating IL-23 with Th17/Treg-associated immune cell activation. Additionally, IL-23 displayed a positive association with MMP-1 and VEGF, implying that IL-23 may be involved in vascular endothelial activation and remodeling in KD.

**Table 3:** Comparative analyses of the associations between circulating IL-23 concentrations and Pro-inflammatory mediators.

Pro-inflammatory mediators	IL-23	
	r	*p*
WBC (pg/mL)	0.17	0.043
CRP (pg/mL)	0.24	0.025
IL-6 (pg/mL)	0.35	0.045
IL-10 (pg/mL)	0.51	0.002
IL-17A (pg/mL)	0.42	0.036
MMP-1 (pg/mL)	0.33	0.013
VEGF (pg/mL)	0.60	0.002

IL-23, interleukin-23; WBC, white blood cell count; CRP, C-reactive protein; IL-6, interleukin-6; IL-10, interleukin-10; IL-17A, interleukin-17A; MMP-1, matrix metalloproteinase-1; VEGF, vascular endothelial growth factor; r, correlation coefficient; *p*, Two-tailed *p*-value.

### Predictive value of circulating IL-23 for KD and CAL development in KD patients

**Figure 4:**
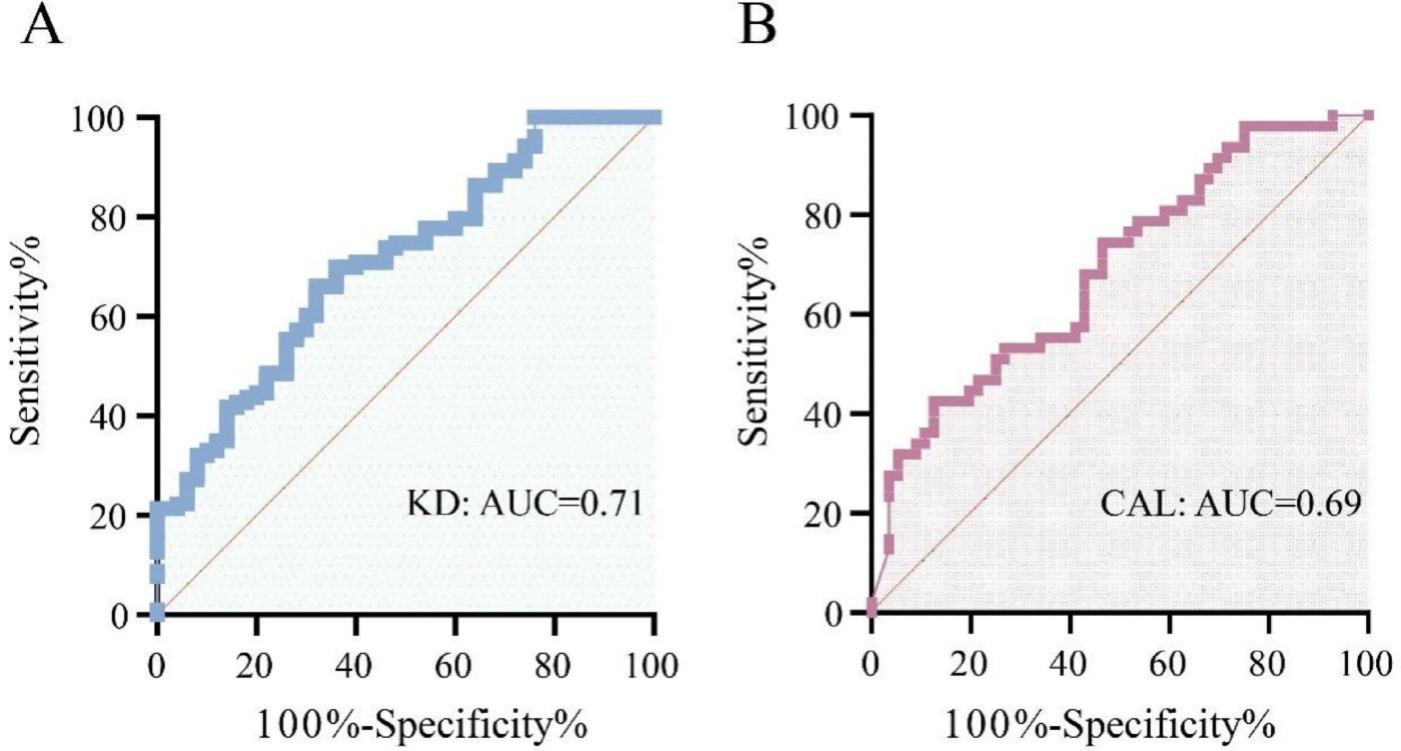
ROC curves of IL-23. **(A)** Assessment of circulating il-23 for the diagnostic discrimination of Kawasaki disease (KD) patients from healthy control subjects. **(B)** Assessment of circulating il-23 for the diagnostic differentiation of KD-CAL from KD-nCAL.

We conducted receiver operating characteristic (ROC) curve analyses to assess the diagnostic utility of circulating IL-23 concentrations for KD (Figure 4). As depicted in Figure 4A, circulating IL-23 concentrations successfully discriminated between pediatric patients with KD and NC, with the curve yielding an area under the ROC curve (AUC) of 0.71 (95% CI: 0.62–0.79, *P* < 0.0001). The optimal threshold value identified via the Youden index was 202.3 pg/mL, with this cutoff value demonstrating 66.02% sensitivity and 68.00% specificity, alongside a positive likelihood ratio (+LR) of 2.06. Using this threshold value, 68 true positive cases, 35 false negative cases, 16 false positive cases, and 34 true negative cases were observed (Table 4). Additionally, to evaluate the relationship between circulating IL-23 concentrations and coronary artery involvement, ROC curve analysis was performed to compare KD-CAL and KD-nCALs (Figure 4B). The resulting AUC was 0.69 (95% confidence interval, CI: 0.59–0.79, *P* = 0.001), denoting a moderate capacity for discrimination between the two KD subgroups. An optimal threshold of 661.2 pg/mL resulted in 42.55% sensitivity and 87.50% specificity, with a + LR of 3.40. At this cutoff value, 20 true positive cases, 27 false negative cases, 7 false positive cases, and 49 true negative cases were documented (Table 4).

**Table 4:** Diagnostic efficiency and confusion matrix analysis of serum IL-23 in differentiating KD and KD-CAL subgroups.

Comparison group	Cut-off (pg/mL)	AUC (95% CI)	Sensitivity (%) (95% CI)	Specificity (%) (95% CI)	TP	FN	FP	TN	+LR
KD vs. control	202.3	0.71 (0.62–0.79)	66.02 (56.44–74.44)	68.00 (54.19–79.24)	68	35	16	34	2.06
KD-CAL vs. KD-nCAL	661.2	0.69 (0.59–0.79)	42.55 (29.51–56.72)	87.50 (76.37–93.81)	20	27	7	49	3.4

## Discussion

IL-23, a recently identified component of the IL-12 cytokine family, has garnered growing recognition as a key regulatory mediator in autoimmune and inflammatory conditions. In the present study, we explored the role played by this cytokine in the immunopathogenic processes underlying KD and evaluated its association in relation to CALs. Three principal observations emerged from our analyses: (1) Circulating IL-23 concentrations exhibited marked upregulation in children diagnosed with KD when compared against both NC participants as well as FC; (2) Circulating IL-23 concentrations showed a considerable increase among children with KD who developed CALs in contrast to KD-affected children without such lesions; and (3) circulating IL-23 concentrations exhibited positive associations with a panel of pro-inflammatory and vascular-associated mediators, encompassing WBC, CRP, IL-6, IL-17A, IL-10, MMP-1, and VEGF. Together, these results implicate IL-23 as a potentially important cytokine in acute KD-related vascular inflammation. However, it should be noted that although this study confirmed significantly higher circulating IL-23 levels in KD patients compared to febrile and healthy controls, it did not systematically compare the differences of other pro-inflammatory cytokines (such as IL-1β, TNF-α, etc.) across the groups. Therefore, the core finding of this study more strongly suggests that elevated IL-23 is closely associated with KD, particularly KD complicated by CAL, and its level demonstrates certain specificity in distinguishing whether KD is accompanied by CAL.

Activated dendritic cells and macrophages represent the primary cellular producers of IL-23, with this cytokine performing a core function in chronic inflammatory pathologies and autoimmune diseases, such as rheumatoid arthritis, asthma, and atherosclerosis (10, 11). Additionally, IL-23 sustains inflammatory processes via macrophage activation, eliciting the secretion of IL-1, TNF-α, and IL-23 *per se* (12, 13). Despite its well-established role in autoimmune vasculitides, studies examining IL-23 in KD remain limited. Our findings confirm significantly elevated IL-23 levels in acute KD, suggesting IL-23 may contribute to vascular inflammation characteristic of this disease. Notably, elevated circulating IL-23 concentrations in KD-CALs further support its possible involvement in coronary artery pathology.

Immune activation mediated by Th17 cells has been identified as a key mediator of inflammatory responses during the acute stage of KD (5, 16, 17). Th17 cells produce and release IL-17, IL-6, and TNF-α—factors that induce endothelial cell activation, extracellular matrix degradation, and tissue damage. In particular, IL-17 elicits robust pro-inflammatory activity through the induction of cytokines, chemokines, and matrix metalloproteinases ^(^18, 19). A multitude of prior investigations have demonstrated that circulating IL-17 concentrations are markedly upregulated in pediatric patients with acute KD (17, 20). At a mechanistic level, IL-23 induces the differentiation and proliferation of Th17 lymphocytes; these lymphocytes then synthesize and secrete IL-17, IL-6, IL-22, TNF-α, and granulocyte-macrophage colony-stimulating factor, thus augmenting inflammatory cascades (12). Our research yielded highly analogous findings, with circulating IL-23 concentrations displaying a positive association with IL-17 and IL-6 concentrations. Such observations suggest that the IL-23/IL-17 signaling axis could be involved in the pathogenetic processes underlying KD.

IL-10 serves as a central immunoregulatory signaling molecule that harbors strong anti-inflammatory characteristics and powerful immunosuppressive capabilities. This molecule curtails the synthesis of major pro-inflammatory mediators, including TNF-α, IL-6, and IL-1β, and exerts a pivotal role in immune tolerance alongside the pathogenetic mechanisms of autoimmune diseases (21–23) Growing lines of evidence demonstrate that IL-10 is involved in the pathogenetic processes of KD. Specifically, IL-10 delivery facilitated via adeno-associated viral (AAV) vectors abrogates vasculitic inflammatory reactions, fibrotic remodeling, and mortality within a murine model of KD (24, 25). Furthermore, multiple investigations have documented that IL-23 augments IL-10 synthesis, which is consistent with our finding that circulating IL-23 concentrations exhibit a positive correlation with IL-10 levels in KD patients (26, 27). Collectively, these observations indicate that IL-23 could induce compensatory IL-10 overexpression during the acute inflammatory phase of KD, acting as part of a self-regulatory mechanism to counterbalance the inflammatory response.

Furthermore, our analyses revealed positive associations between circulating IL-23 concentrations and WBC count, CRP levels, MMP-1, and VEGF among children with KD. MMP-1, belonging to the CC-chemokine family responsible for monocyte recruitment and activation, is involved in vascular inflammatory damage and exhibits a positive correlation with the progression of KD (28–30). Although evidence on the IL-23–MMP-1 axis remains limited, our results suggest that IL-23 may promote MMP-1 expression, thereby contributing to vascular inflammation in acute KD. Likewise, increased WBC counts and CRP levels are well-recognized indicators of systemic inflammatory responses, with CRP being identified as an independent predictive factor for giant coronary aneurysms in individuals diagnosed with KD (31). VEGF, a potent endothelial mitogen, increases vascular permeability and is considered a predictor of coronary arteritis lesions in KD (32–35) Consistent with prior reports that IL-23 promotes VEGF production in malignant tumors (27, 36), we identified a strong association between IL-23 and VEGF levels in KD. Collectively, these correlations support a mechanistic hypothesis in which IL-23 amplifies vascular inflammation and endothelial activation, thereby contributing to coronary artery lesion formation in KD. Additionally, this study found that IL-23 showed positive correlations with commonly used clinical inflammatory markers such as ESR, WBC count, and CRP. This association suggests that combining IL-23 with these routine indicators for analysis might enable the construction of a more effective risk assessment model. In the future, it is worth exploring the practical value of their combined application in predicting the occurrence of coronary artery lesions. It is particularly noteworthy that this study found serum IL-23 exhibited a high specificity (87.5%) in distinguishing KD-nCAL from KD-CAL patients. This indicates that the proposed cut-off value (661.2 pg/mL) demonstrates good efficacy in identifying KD patients unlikely to develop coronary artery lesions (i.e., CAL-negative patients). High specificity implies a low false-positive rate, which holds significant value for clinical decision-making. For example, in children with IL-23 levels below this threshold, clinicians may have greater confidence that the risk of developing CAL is relatively low, potentially allowing for individualized adjustments to treatment strategies under close monitoring. This enhances the potential clinical relevance of IL-23 as a predictive biomarker.

IVIG in conjunction with aspirin constitutes the contemporary first-line therapy for KD, a regimen that exerts a significant effect in alleviating systemic inflammatory processes and mitigating coronary arterial abnormalities. Second-line interventions for refractory KD have been defined as adjunctive therapeutic methods, which include corticosteroid agents, infliximab, cyclosporine A, anakinra, and plasma exchange procedures (37). As a monoclonal antibody-based agent directed against the IL-23 p19 subunit, risankizumab has shown therapeutic activity in managing autoimmune conditions like psoriasis and ulcerative colitis (38, 39). Given our findings that IL-23 is associated with multiple inflammatory mediators and vascular injury markers, IL-23 blockade may represent a rational therapeutic strategy in KD, particularly for preventing CALs (40). Therefore, anti-IL-23 agents warrant further investigation as potential targeted therapies among high-risk individuals diagnosed with KD.

In this investigation, we conducted a concurrent assessment of how IL-23 performs in terms of predictive utility within two clinically different contexts—the diagnostic confirmation of KD and the prediction of CALs—within the same patient cohort. IL-23 demonstrated moderate discriminative capacity for KD (AUC = 0.71, *P* < 0.0001), supporting its potential as an adjunct biomarker to current diagnostic criteria. Conversely, its ability to predict CALs was relatively modest (AUC = 0.69, *P* = 0.001), although the trend suggests possible early-warning value for vascular involvement. Therefore, circulating IL-23 levels may serve dual clinical functions: assisting in disease identification and signaling heightened risk for coronary artery complications. These findings highlight IL-23 as a candidate laboratory indicator to inform refined, stratified management strategies in KD.

However, this study also has several limitations that should be considered when interpreting the results. First, as noted above, this study did not perform parallel comparisons of the levels of other key pro-inflammatory cytokines between the KD group and the control groups. Therefore, it cannot be claimed that IL-23 possesses unique specificity compared to other inflammatory mediators in the diagnosis of KD. Our conclusions should instead be interpreted as focusing more on its association with the risk of coronary artery lesions. Second, the observed correlation between IL-23 and white blood cell count was relatively weak (r = 0.17), and its biological and clinical significance requires further validation through studies with larger sample sizes. Additionally, the observed association between IL-23 and white blood cell count was relatively weak (r = 0.17), and its biological and clinical significance requires further validation in studies with larger sample sizes. Moreover, the primary focus of this study was on the correlation between circulating IL-23 and laboratory-based inflammatory and vascular markers. It did not systematically collect or analyze granular data on individualized detailed clinical symptom profiles (such as rash, conjunctival injection, or extremity changes) in children with Kawasaki disease. Consequently, this study was unable to evaluate the association between IL-23 levels and specific clinical features. This limitation somewhat restricts the direct linkage between laboratory findings and clinical symptoms, and the clinical applicability of these results needs further validation in future studies integrating detailed phenotypic data. Second, this study is a single-center observational investigation with a limited sample size, particularly in subgroup analyses, which may affect statistical power and limit the generalizability of the findings to broader populations. Additionally, all samples were collected before IVIG treatment and were measured at a single time point, failing to dynamically reflect the changes in IL-23 levels throughout the course of the disease and after treatment. Although cytokines were measured using standard ELISA methods, their concentrations may be influenced by individual variations and pre-processing factors. Finally, this study primarily focused on IL-23 and related inflammatory markers. Future research should integrate multi-omics data to more comprehensively elucidate the molecular mechanisms underlying vascular lesions in KD. These limitations suggest that the current conclusions are preliminary, highlighting the need for prospective, multicenter, longitudinal studies to further confirm the specific role of IL-23 in KD coronary artery lesions and its potential clinical translation.

To summarize, circulating IL-23 levels were markedly upregulated in individuals diagnosed with KD, especially among individuals with concurrent CALs. Furthermore, during the acute stage of KD, circulating IL-23 concentrations exhibited positive associations with core inflammatory cytokines, encompassing IL-6, IL-17A, IL-10, MMP-1, and VEGF. Taken together, the findings indicate that IL-23 could contribute pathologically to the inflammatory cascades that drive vasculitic damage in KD. Additional mechanistic investigations are warranted to define the precise function of IL-23 in vascular pathological processes associated with KD, as well as to evaluate the potential therapeutic efficacy of IL-23 neutralization in this pediatric vasculitic disorder.

## Data Availability

The raw data supporting the conclusions of this article will be made available by the authors, without undue reservation.
